# Urogenitale Beschwerden bei Radfahrern und Reduktion der perinealen Druckbelastung durch einen prostatavolumenadaptierten Fahrradsattel

**DOI:** 10.1055/a-2879-3182

**Published:** 2026-06-09

**Authors:** Horst Hohmuth, Christoph Möller

**Affiliations:** 1 Urologie Andrologie SportmedizinUropraxis UlmUlmGermany; 2 Medizinische FakultätUniversität UlmUlmGermany

**Keywords:** Prostata, Radfahren, Perineum, Genitalbeschwerden, Fahrradsattel, prostate, perineum, bicycle saddle, cycling, urogenital symptoms

## Abstract

**Hintergrund:**

Urogenitale Beschwerden wie perinealer Druckschmerz, penile Taubheit oder Miktionsbeschwerden im Zusammenhang mit dem Fahrradfahren werden im klinischen Alltag urologischer Praxen regelmäßig berichtet und können insbesondere bei vorerkrankten Patienten klinische Relevanz besitzen.

**Ziel der Arbeit (Fragestellung):**

Ziel der vorliegenden Studie war es, die Prävalenz von radfahrassoziierten Beschwerden in einer urologischen Kohorte zu erfassen und die perineale Druckverteilung unter Verwendung eines prostatavolumenadaptierten Fahrradsattels (Prostasella) zu untersuchen. Zusätzlich wurde ein explorativer Algorithmus zur nicht-invasiven Abschätzung des Prostatavolumens entwickelt, um eine patientenspezifische Sattelgrößenbestimmung zu ermöglichen.

**Material und Methoden:**

Insgesamt wurden 50 männliche Patienten einer urologischen Praxis klinisch untersucht und standardisiert befragt. Das Prostatavolumen wurde mittels transrektaler Sonographie bestimmt. Die sensorbasierte perineale Druckmessung erfolgte in einer Subkohorte von 24 Patienten. Hierbei wurden zwei voneinander unabhängige Gruppen untersucht: eine Interventionsgruppe unter Verwendung eines prostatavolumenadaptierten Fahrradsattels sowie eine Kontrollgruppe unter Verwendung eines konventionellen Fahrradsattels unter vergleichbaren Messbedingungen. Der Vergleich der zentralen perinealen Druckbelastung erfolgte als unabhängiger Gruppenvergleich und nicht als intraindividueller Crossover-Vergleich. Primärer Endpunkt war die zentrale perineale Druckbelastung im definierten perinealen Messbereich.

**Ergebnisse:**

Die Verwendung des prostatavolumenadaptierten Fahrradsattels führte zu einer signifikanten Reduktion der zentralen perinealen Druckbelastung im Vergleich zu konventionellen Sattelsystemen (Reduktion um ca. 88 %, *P* < 0,001).

**Schlussfolgerung:**

Eine prostatavolumenadaptierte Sattelkonstruktion ermöglicht eine relevante Reduktion der perinealen Druckbelastung und stellt einen potenziell klinisch relevanten Ansatz zur Prävention und Therapie radfahrassoziierter urogenitaler Beschwerden dar. Der entwickelte Algorithmus ist als explorativer Ansatz zu werten und bedarf weiterer Validierung.

## Abstract (en)

**Background:**

Urogenital symptoms associated with cycling, such as perineal pain, penile numbness, or lower urinary tract symptoms, are frequently reported in routine urological practice and may be of particular clinical relevance in predisposed patients.

**Objectives:**

The aim of this study was to assess the prevalence of cycling-associated symptoms in a urological cohort and to investigate perineal pressure distribution using a prostate volume-adapted bicycle saddle (Prostasella). In addition, an exploratory algorithm for non-invasive estimation of prostate volume was developed to enable individualized saddle sizing.

**Materials and Methods:**

A total of 50 male patients from a urological practice underwent a clinical examination and standardized assessment of cycling-associated symptoms. Prostate volume was determined by transrectal ultrasound. Sensor-based perineal pressure measurements were performed in a subgroup of 24 patients. Two independent groups were evaluated: an intervention group using a prostate volume-adapted bicycle saddle and a control group using a conventional bicycle saddle under comparable measurement conditions. Central perineal pressure load was compared between independent groups rather than in an intra-individual crossover design. The primary endpoint was central perineal pressure load within the predefined perineal measurement area.

**Results:**

The use of a prostate volume-adapted bicycle saddle resulted in a significant reduction in central perineal pressure load compared with conventional saddle systems (reduction of approximately 88%, *P* < 0.001).

**Conclusions:**

A prostate volume-adapted saddle design enables a clinically relevant reduction in central perineal pressure load and represents a potentially relevant approach for the prevention and treatment of cycling-associated urogenital symptoms. The developed algorithm should be considered exploratory and requires further validation.

Radfahren ist als Ausdauer- und Alltagsaktivität weit verbreitet und gilt als gesundheitsfördernd. Gleichzeitig treten bei männlichen Radfahrern häufig perineale und urogenitale Beschwerden auf, die auf eine anhaltende Druckbelastung im Dammbereich zurückgeführt werden. Insbesondere bei urologisch vorbelasteten Patienten könnte deshalb eine Anpassung der Sattelgeometrie unter Bezugnahme auf Prostatavolumen oder -operationen erforderlich sein.

## Einleitung

Radfahren gilt als gelenkschonende aerobe Trainingsform mit nachgewiesenen positiven Effekten auf kardiovaskuläre und metabolische Parameter, ist jedoch auch mit charakteristischen sportartspezifischen Verletzungsmustern assoziiert 1 .

In der urologischen Praxis ist diesbezüglich das Auftreten urogenitaler Beschwerden bei männlichen Radfahrern im Zusammenhang mit dem Radsport ein relevantes Thema. Klinisch manifestieren sich diese als perineale Schmerzen, peniles Taubheitsgefühl, erektile Dysfunktion oder irritative Miktionsbeschwerden, darüber hinaus werden potenzielle Auswirkungen auf die männliche Fertilität diskutiert 2 3 4 5 6 . Während des Pedalierens wird ein erheblicher Anteil des Körpergewichts über den Sattel auf den Damm übertragen. Dabei können neurovaskuläre Strukturen mechanisch komprimiert werden. In der Literatur werden lokale Ischämien und ischämisch bedingte Neuropathien des Penis, Nervenkompressionen bis hin zu Pudendusneuropathien und -verletzungen sowie vaskuläre Komplikationen beschrieben. Zudem konnte eine signifikante Reduktion des penilen Sauerstoffpartialdrucks in Abhängigkeit vom Satteltyp nachgewiesen werden 6 7 8 9 10 11 12 13 14 .

Die berichtete Prävalenz perinealer Beschwerden reicht von 7 % bis 61 %, die einer erektilen Dysfunktion von 4 % bis 19 % 3 15 16 17 18 19 20 . Mehrere Studien zeigen signifikante Zusammenhänge zwischen Radfahren sowie sexueller und urologischer Funktion 18 . Darüber hinaus belegen Untersuchungen nach Langstrecken-Radrennen signifikante Zusammenhänge zwischen erektiler Dysfunktion und spezifischen Fahrrad- bzw. Sattelmerkmalen 15 . Dies ist bereits in früheren Untersuchungen im Hinblick auf urologische Erkrankungen im Zusammenhang mit Langstrecken-Radrennen beschrieben worden 16 .

Neben den bereits beschriebenen perinealen Beschwerden werden im Zusammenhang mit dem Radfahren weitere funktionelle und neurologische Symptome berichtet. Hierzu zählen insbesondere Sensibilitätsstörungen im Genitalbereich, Dysästhesien sowie funktionelle Einschränkungen der Sexualfunktion. Auch irritative und obstruktive Miktionsbeschwerden können im Kontext chronischer perinealer Druckbelastung auftreten. Aktuelle deutschsprachige Übersichtsarbeiten unterstreichen die klinische Relevanz dieser Beschwerdebilder und deren Zusammenhang mit der individuellen Belastungssituation beim Radfahren 21 22 .

Etablierte ergonomische Sattelkonzepte zielen primär auf eine Druckverlagerung auf die Sitzbeinhöcker ab, wobei sowohl die Rumpfposition als auch das Satteldesign einen signifikanten Einfluss auf die perineale Druckverteilung haben 20 . Die altersassoziierte Zunahme des Prostatavolumens als potenziell druckrelevanter Faktor im kleinen Becken wird hierbei bislang nicht systematisch berücksichtigt. Insbesondere bei Patienten mit benignem Prostatasyndrom oder nach operativen Eingriffen an der Prostata kann eine zentrale perineale Druckbelastung klinisch relevante Beschwerden verstärken. Eine individualisierte Anpassung der Sattelgeometrie an das Prostatavolumen ist bislang nicht etabliert. Vor diesem Hintergrund wurde ein prostatavolumenadaptierter Fahrradsattel entwickelt, der eine gezielte Entlastung des zentralen perinealen Bereichs ermöglichen soll.

Ziel der vorliegenden Studie war es, die Häufigkeit und klinische Relevanz radfahrassoziierter urogenitaler Beschwerden bei urologischen Patienten zu erfassen, die perineale Druckverteilung unter Verwendung eines prostatavolumenadaptierten Fahrradsattels zu analysieren und zu prüfen, ob hierdurch eine signifikante Reduktion der zentralen perinealen Druckbelastung im Vergleich zu konventionellen Sattelmodellen erzielt werden kann. Zusätzlich wurde ein praxistauglicher Algorithmus zur nicht-invasiven Approximation des Prostatavolumens entwickelt, um eine patientenspezifische Sattelgrößenbestimmung zu ermöglichen.

## Material und Methoden

### Studiendesign

Es handelt sich um eine prospektive klinische Beobachtungs- und Validierungsstudie zur Untersuchung radfahrassoziierter urogenitaler Beschwerden sowie der perinealen Druckverteilung unter Verwendung eines prostatavolumenadaptierten Fahrradsattels. Die klinische Datenerhebung erfolgte in einer urologischen Praxis.

Die biomechanische Druckmessung wurde als vergleichende Teiluntersuchung mit zwei voneinander unabhängigen Messgruppen durchgeführt. Eine Interventionsgruppe wurde unter Verwendung des prostatavolumenadaptierten Sattels untersucht, während eine separate Kontrollgruppe unter vergleichbaren Messbedingungen auf einem konventionellen Fahrradsattel gemessen wurde. Es handelte sich somit nicht um einen intraindividuellen Crossover-Vergleich, sondern um einen unabhängigen Gruppenvergleich.

### Studienpopulation

Insgesamt wurden 50 männliche Patienten im Alter von 40–85 Jahren prospektiv in die Studie eingeschlossen. Voraussetzung war eine regelmäßige Radfahraktivität von mindestens 500 km pro Jahr.

Bei allen Studienteilnehmern erfolgte eine standardisierte Erhebung radfahrassoziierter urogenitaler Beschwerden sowie die sonographische Bestimmung des Prostatavolumens. Zusätzlich wurden demografische Parameter und urologische Vorerkrankungen erfasst.

Die sensorgestützte biomechanische Druckmessung wurde aufgrund methodischer Limitationen als validierende Teiluntersuchung in einer Subkohorte von 24 Patienten durchgeführt. Ergänzend wurde eine unabhängige Kontrollgruppe von 45 Probanden unter vergleichbaren Messbedingungen auf einem konventionellen Fahrradsattel untersucht. Diese Kontrollgruppe diente ausschließlich dem biomechanischen Vergleich der zentralen perinealen Druckbelastung und war nicht Teil der klinischen Hauptkohorte.

Damit beziehen sich die klinisch-urologischen Angaben auf die Gesamtkohorte (n = 50), während sich die biomechanische Analyse auf zwei unabhängige Messgruppen stützt: eine Interventionsgruppe (n = 24) und eine Kontrollgruppe (n = 45).

Einschlusskriterien waren eine dokumentierte urologische Vorgeschichte (z. B. benigne Prostatahyperplasie oder Zustand nach Prostataoperation), die schriftliche Einwilligung zur Studienteilnahme sowie die vollständige Durchführung aller Untersuchungen. Ausschlusskriterien umfassten akute urologische Infektionen, bekannte maligne Erkrankungen des Urogenitaltrakts, neurologische Erkrankungen mit Einfluss auf die Blasenfunktion, relevante orthopädische Erkrankungen mit möglicher Beeinflussung der Druckmessung sowie unvollständige Datensätze.

Eine Übersicht der demografischen, klinischen und radfahrassoziierten Charakteristika der Studienpopulation ist in Tab. 1 zusammengefasst.

**Table:** **Tab. 1** Charakteristika der Studienpopulation (n = 50).

**Parameter**	**Wert**
**Demografische Daten**	
Alter (Jahre)	66
Körpergröße (cm)	178
Körpergewicht (kg)	82
Prostatavolumen (cm³)	39
**Radfahrverhalten**	
Radfahrdauer (Jahre)	38
Durchschnittliche Fahrleistung (km/Jahr)	2877
**Urogenitale Beschwerden**	
Probleme mit dem Sattel	26 (52 %)
Urogenitale Beschwerden insgesamt	38 (76 %)
Nachträufeln	8 (16 %)
Häufiger Harndrang	17 (34 %)
Vermehrtes Wasserlassen	26 (52 %)
**Prostataanamnese**	
Bekannte Prostataerkrankung	19 (38 %)
Zustand nach Prostataoperation	10 (20 %)
Medikamentös behandelt	9 (18 %)

### Datenerhebung

Das Prostatavolumen wurde bei allen Studienteilnehmern mittels transrektaler Sonographie (TRUS) bestimmt.

Die perineale Druckverteilung wurde mithilfe eines sensorgestützten Fahrradsattels (Sensortechnik: gebioMized, Pressure Mapping System GPMS) unter standardisierten Bedingungen in einem kontrollierten Messaufbau objektiv erfasst. Die Messungen erfolgten auf einem stationären Testfahrrad.

Alle Probanden wurden unter vergleichbaren Sitzbedingungen untersucht. Die Sitzposition entsprach einer typischen, leicht nach vorne geneigten Oberkörperhaltung, wie sie bei der Nutzung eines Fahrrads im Alltag üblich ist. Die Füße befanden sich während der Messung auf den Pedalen, um eine realitätsnahe Belastungssituation zu simulieren.

Die Druckmessung erfolgte über einen definierten Messzeitraum unter kontinuierlicher Belastung. Ziel war eine möglichst standardisierte Erfassung der Druckverteilung bei gleichbleibenden Versuchsbedingungen. Individuelle Unterschiede im Sitzverhalten konnten aufgrund des explorativen Studiendesigns nicht vollständig standardisiert werden, wurden jedoch durch die einheitliche Versuchsanordnung minimiert.

**Abb. 1:**
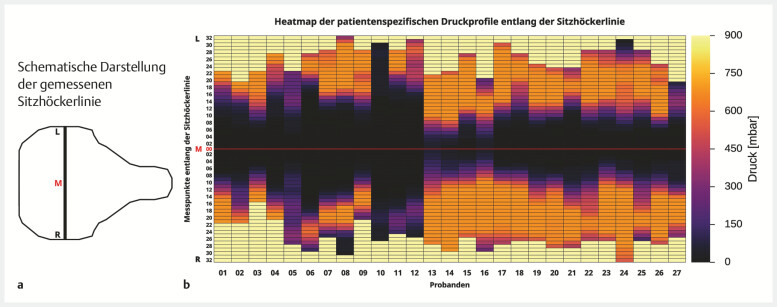
Farbkodierte Heatmap der perinealen Druckverteilung unter Verwendung des prostatavolumenadaptierten Sattels. Warme Farben (rot/orange) kennzeichnen Bereiche erhöhter Druckbelastung, während kühle Farben (blau/grün) Bereiche geringerer Druckbelastung anzeigen. Erkennbar ist eine Entlastung im zentralen perinealen Bereich bei gleichzeitiger lateraler Druckverlagerung auf die Sitzbeinhöcker.

Die Drucksensoren waren in die Satteloberfläche integriert und ermöglichten eine hochauflösende Erfassung der perinealen Druckverteilung. Die Datenauswertung erfolgte softwaregestützt. Primärer Messparameter war die zentrale perineale Druckbelastung im Bereich der Sattelaussparung ( Abb. 1 ). Zusätzlich wurde die Druckverteilung entlang der Sitzhöckerachse analysiert.

Die Druckverteilung wurde als farbkodierte Heatmap visualisiert, wobei höhere Druckwerte durch warme Farbtöne (rot/orange) und niedrigere Druckwerte durch kühle Farbtöne (blau/grün) dargestellt wurden.

Die biomechanische Druckmessung wurde als validierende Teiluntersuchung in einer ausgewählten Subkohorte durchgeführt, was die in den Ergebnissen dargestellten unterschiedlichen Fallzahlen erklärt.

Zusätzlich wurden Alter, Körpergröße und Körpergewicht sowie anamnestische Angaben zu radfahrassoziierten urogenitalen Beschwerden erhoben. Eine standardisierte Sportanamnese dokumentierte Fahrleistung, Trainingsfrequenz und Einsatzbereich.

Da die Messungen in zwei unabhängigen Gruppen durchgeführt wurden, lagen keine gepaarten Messwerte vor. Der Vergleich zwischen konventionellem Fahrradsattel und prostatavolumenadaptiertem Sattel wurde daher als unabhängiger Gruppenvergleich ausgewertet.

### Algorithmische Schätzung des Prostatavolumens

**Abb. 2:**
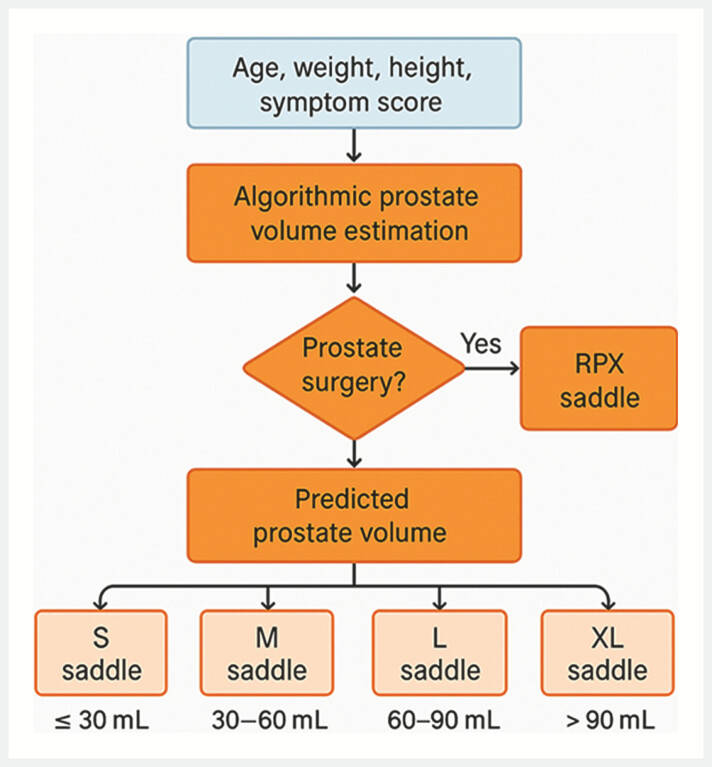
Schematische Darstellung des Algorithmus zur Volumenabschätzung und Sattelgrößenklassifikation.

Zur nicht-invasiven Approximation des Prostatavolumens wurde ein praxistauglicher Algorithmus entwickelt. Ziel war nicht die exakte volumetrische Vorhersage, sondern die Einteilung in klinisch relevante Größenklassen als Grundlage für eine orientierende Sattelgrößenempfehlung ( Abb. 2 ).

Das Modell basierte auf klinisch leicht zugänglichen Parametern (Alter, Körpergröße, Körpergewicht sowie einem standardisierten urogenitalen Beschwerdescore). Die Symptomangaben wurden in eine harmonisierte ordinale Skala überführt.

Die algorithmische Modellierung erfolgte unter Einsatz eines Machine-Learning-basierten Klassifikationsverfahrens. Aufgrund der begrenzten Fallzahl und der ungleichen Verteilung der Volumenklassen wurde ein Random-Forest-Klassifikator verwendet, um potenziell nichtlineare Zusammenhänge abzubilden. Zur Reduktion von Verzerrungen durch Klassenimbalance wurde eine gewichtete Modellierung implementiert.

Die Modellvalidierung erfolgte im Rahmen eines Train-Test-Splits bei kleiner Stichprobe und ist daher als explorativ zu bewerten. Die erzielte Klassifikationsgenauigkeit lag im moderaten Bereich, wobei eine exakte volumetrische Vorhersage nur eingeschränkt möglich war.

Der entwickelte Algorithmus ist daher primär als orientierendes Klassifikationsmodell zur Einteilung des Prostatavolumens in klinisch relevante Größenklassen zu verstehen und nicht als diagnostisches Verfahren geeignet.

Eine Übertragung in die klinische Anwendung erfordert eine externe Validierung in unabhängigen Kohorten.

### Ergebnisse

In der untersuchten Kohorte lag das mittlere Alter bei 66 Jahren, das mittlere sonographisch bestimmte Prostatavolumen bei 39 ml. 38 % der Patienten wiesen eine bekannte Prostataerkrankung auf, wobei 52 % dieser Patienten operativ vorbehandelt waren. Mehr als die Hälfte der Studienteilnehmer (52 %) berichtete über radfahrassoziierte urogenitale Beschwerden unter Nutzung konventioneller Sättel.

Bei 24 Probanden wurde eine biomechanische Druckmessung auf dem prostatavolumenadaptierten Sattelmodell durchgeführt. Bei 45 Probanden erfolgte die Druckmessung auf einem konventionellen Sattelmodell. Aufgrund technischer Artefakte konnten 23 Datensätze in die finale Analyse einbezogen werden.

Die demografischen und klinischen Basischarakteristika der Messkohorten unterschieden sich nicht signifikant von der Gesamtkohorte.

**Abb. 3:**
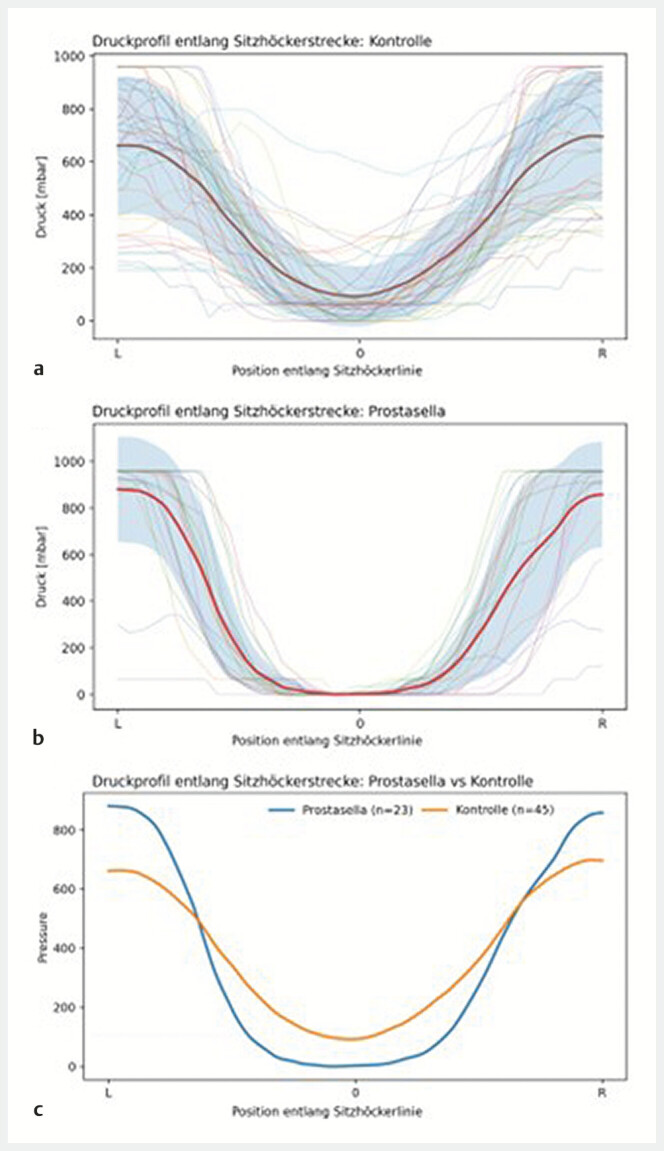
Graphischer Vergleich der perinealen Druckbelastung zwischen ( **a** ) konventionellem Sattel und, ( **b** ) prostatavolumenadaptiertem Sattel entlang der Sitzhöckerachse, sowie ( **c** ) Vergleich der Mittelwertskurven beider Varianten. **a,b** Die Mittelwertskurven sind in rot und die Standardabweichung in hellblau hervorgehoben. Zur übersichtlichen Visualisierung wurden alle Druckverlaufskurven auf dieselbe Distanz zwischen den Sitzhöckern normalisiert. Es kann ein deutlicher Unterschied der Druckbelastung im Bereich des Perineums zwischen beiden Sattelvarianten beobachtet werden.

Unter Verwendung konventioneller Sättel betrug der mittlere zentrale perineale Druck 179,7 ± 148,6 mbar (n = 45), während dieser unter dem prostatavolumenadaptierten Sattel bei 20,9 ± 25,3 mbar (n = 23) lag ( Abb. 3 ).

Dies entspricht einer absoluten Differenz von 158,8 mbar (95 %-Konfidenzintervall 113,0–204,6 mbar) sowie einer relativen Reduktion von 88 %.

Der Gruppenunterschied war hochsignifikant (Welch-t-Test, p < 0,001) und wurde durch einen nichtparametrischen Wilcoxon-Rangsummentest bestätigt (p < 0,001). Die Effektstärke war groß (Hedges’ g = 1,28). Die stochastische Dominanzanalyse ergab einen Cliff’s δ von 0,91 (95 %-KI 0,79–0,97).

**Abb. 4:**
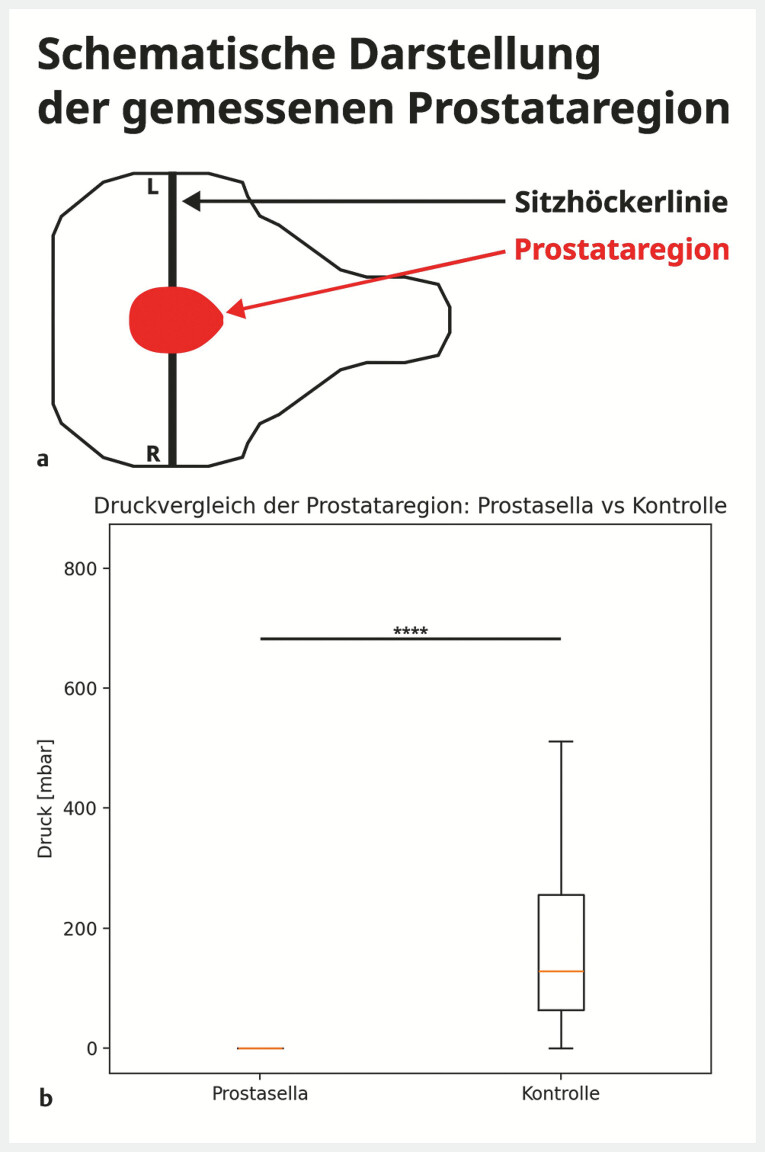
Statistischer Vergleich der zentralen perinealen Druckbelastung zwischen konventionellem Sattel und prostatavolumenadaptiertem Sattel. Abgebildet sind die Werte aller Druckmessungspunkte des perinealen Bereichs jedes Probanden.

Die visuelle Vergleichsdarstellung der Druckverteilung verdeutlichte eine zentrale Entlastung im Bereich der Sattelaussparung sowie eine laterale Druckumverteilung auf die Sitzbeinhöcker unter Verwendung des volumenadaptierten Satteldesigns ( Abb. 4 ).

### Biologische Baseline

Zur deskriptiven Charakterisierung wurde die Beziehung zwischen Prostatavolumen und verfügbaren Kovariaten analysiert. In der Kohorte (n = 50) zeigte sich eine deutlich schiefe Verteilung mit Überrepräsentation kleiner Volumina (< 30 ml; n = 29) gegenüber mittleren (30–60 ml; n = 16) und großen Volumina (> 60 ml; n = 5).

Alter und BMI korrelierten erwartungsgemäß positiv mit dem Prostatavolumen, erklärten die Varianz jedoch nur teilweise. Subjektive Symptomangaben (IPSS/Beschwerde-Score) wurden in eine harmonisierte fünfstufige Skala (0–4) überführt.

Insgesamt zeigte sich eine ausgeprägte Klassenimbalance sowie eine nur moderate Vorhersagbarkeit aus Einzelprädiktoren, was den Einsatz robuster, nichtlinearer Modellierungsansätze erforderlich machte.

### Machine-Learning-Ansatz zur 3-Klassen-Klassifikation

Auf Basis der deskriptiven Analyse wurde ein überwachtes Modell zur Einteilung des Prostatavolumens in drei klinisch relevante Kategorien (< 30 ml, 30–60 ml, > 60 ml) entwickelt. Die Modellierung erfolgte als Volumenklassifikation.

Zur Abbildung potenziell nichtlinearer Zusammenhänge wurde ein Random-Forest-Klassifikator (ranger) eingesetzt. Das Feature-Set umfasste Alter, BMI und den harmonisierten Symptomindex. Aufgrund der Klassenimbalance wurde eine klassenbasierte Gewichtung verwendet.

Die Modellauswahl erfolgte mittels Kreuzvalidierung unter Verwendung von Balanced Accuracy und Macro-F1 als Leistungsmetriken. Im unabhängigen Testsplit (Train: n = 40; Test: n = 10) erreichte das finale Modell eine Accuracy von 0,62. Ergänzend ergaben sich für die kontinuierliche Volumenapproximation ein RMSE von 18 ml und ein MAE von 13,7 ml.

Der Machine-Learning-basierte Ansatz ist als explorativ zu verstehen und diente der Hypothesengenerierung zur Abschätzung prostatavolumenadaptierter Sattelparameter. Aufgrund der begrenzten Fallzahl ist eine Validierung in größeren Kollektiven erforderlich.

## Diskussion und Schlussfolgerung

Die vorliegenden Daten unterstreichen die klinische Relevanz radfahrassoziierter perinealer und genitaler Beschwerden bei urologischen Patienten. Mit einer Prävalenz von 52 % symptomatischer Patienten unter Nutzung konventioneller Sättel zeigt sich die Problematik insbesondere in einer urologisch vorbelasteten Kohorte als bedeutsam. Die Reduktion der zentralen perinealen Druckbelastung um 88 % durch ein prostatavolumenadaptiertes Satteldesign war statistisch hochsignifikant und mit einer großen Effektstärke verbunden, was auf einen robusten biomechanischen Entlastungseffekt hinweist.

Die Ergebnisse sind im Kontext der bestehenden Literatur plausibel. Beschrieben werden neurovaskuläre Kompressionsmechanismen, ischämisch bedingte Neuropathien sowie funktionelle Störungen bis hin zur erektilen Dysfunktion infolge perinealer Druckbelastung beim Radfahren 6 7 8 9 10 11 12 13 14 . Insbesondere die Abhängigkeit des penilen Sauerstoffpartialdrucks vom Satteltyp 13 verdeutlicht die pathophysiologische Bedeutung einer zentralen Druckreduktion.

Etablierte ergonomische Konzepte fokussieren primär auf die Druckverlagerung auf die Sitzbeinhöcker 20 , berücksichtigen jedoch individuelle anatomische Parameter wie das Prostatavolumen bislang nicht systematisch. Gerade bei Patienten mit benignem Prostatasyndrom oder nach operativen Eingriffen kann eine persistierende zentrale Belastung symptomverstärkend wirken. Die in dieser Studie nachgewiesene gezielte Entlastung im Bereich der Sattelaussparung führte zu einer klar dokumentierten Druckumverteilung, die sowohl quantitativ als auch visuell nachvollziehbar war.

Eine wesentliche Limitation der vorliegenden Studie besteht darin, dass ausschließlich ein biomechanischer Surrogatparameter (zentrale perineale Druckbelastung) untersucht wurde, ohne Erhebung patientenrelevanter klinischer Endpunkte. Insbesondere konnte nicht geprüft werden, ob die beobachtete Druckreduktion mit einer Verbesserung von Schmerzen, Taubheitsgefühl, Miktionsbeschwerden oder sexuellen Funktionsstörungen einhergeht.

Das gewählte Studiendesign ist vor dem Hintergrund des Entwicklungsstatus des untersuchten Sattels zu sehen. Es handelt sich um einen Prototyp, dessen biomechanische Eigenschaften zunächst unter standardisierten, laborgebundenen Bedingungen evaluiert wurden. Die Messungen erfolgten auf einem sensorgestützten Testsystem und erlaubten keine Beurteilung von Langzeiteffekten unter Alltagsbedingungen.

Die Übertragbarkeit der dargestellten Druckentlastung auf einen klinischen Nutzen kann daher auf Basis der vorliegenden Daten nicht abschließend beurteilt werden. Hierfür sind prospektive, kontrollierte Studien mit patientenrelevanten Endpunkten erforderlich. Eine entsprechende Folgestudie ist nach regulatorischer Zulassung des Sattels geplant. Dabei sollen Patienten den Sattel über einen Zeitraum von 3–6 Monaten unter Alltagsbedingungen verwenden und die Auswirkungen auf urogenitale Beschwerden mittels standardisierter Fragebögen systematisch erfasst werden.

Ein weiterer wesentlicher Aspekt ist der potenzielle Interessenkonflikt. Der Erstautor ist Erfinder und Patentinhaber des untersuchten Satteldesigns sowie Geschäftsführer des entsprechenden Unternehmens. Hieraus ergibt sich grundsätzlich die Möglichkeit einer Beeinflussung von Studiendesign, Datenauswertung und Interpretation der Ergebnisse. Zur Reduktion solcher Verzerrungen wurde die Datenerhebung standardisiert durchgeführt und die Auswertung anhand objektiver, messbasierter Parameter vorgenommen.

Vor diesem Hintergrund sollten die Ergebnisse mit entsprechender Zurückhaltung interpretiert werden. Eine unabhängige externe Validierung in prospektiven, kontrollierten Studien ist erforderlich, um die Reproduzierbarkeit der Befunde sowie eine mögliche klinische Relevanz belastbar zu bestätigen.

Zusammenfassend sind die Ergebnisse der vorliegenden Arbeit als biomechanischer Wirksamkeitsnachweis zu verstehen und zeigen, dass eine prostatavolumenadaptierte Sattelkonstruktion die zentrale perineale Druckbelastung signifikant reduziert und damit eine Grundlage für weiterführende klinische Untersuchungen darstellt. Eine potenzielle klinische Relevanz, insbesondere für urologische Patienten mit Prostataerkrankungen sowie im postoperativen Kontext, ist in zukünftigen Studien zu prüfen.

## Fazit für die Praxis

- Radfahrassoziierte urogenitale Beschwerden sind bei urologischen Patienten häufig und sollten aktiv anamnestisch erfasst werden.
- Eine erhöhte zentrale perineale Druckbelastung stellt einen relevanten biomechanischen Risikofaktor für Schmerzen, Taubheitsgefühl und funktionelle Beschwerden dar.
- Eine ergonomische Sattelgeometrie unter Berücksichtigung des Prostatavolumens kann die zentrale perineale Druckbelastung signifikant reduzieren.
- Bei Patienten mit benignem Prostatasyndrom oder nach Prostataoperation sollte die Sattelkonfiguration gezielt angepasst werden, insbesondere im Rahmen der sportlichen Aktivität und der postoperativen Rehabilitation.

## Ethische Standards

Die vorliegende Untersuchung wurde als prospektive klinische Validierungsstudie eines neu entwickelten, prostatavolumenadaptierten Fahrradsattels durchgeführt. Es handelte sich um eine nicht-interventionelle biomechanische Messstudie ohne therapeutischen Eingriff oder Veränderung bestehender medizinischer Behandlungsstrategien.

Zum Zeitpunkt der Datenerhebung befand sich das Produkt in der Entwicklungsphase und war nicht als Medizinprodukt im Sinne der Medical Device Regulation in Verkehr gebracht. Die Untersuchung erfolgte ausschließlich zur Analyse der Druckverteilung unter praxisnahen Bedingungen.

Nach Prüfung der lokalen regulatorischen Rahmenbedingungen bestand für diese Art der nicht-invasiven, nicht-therapeutischen Messstudie keine verpflichtende Vorlage bei einer Ethikkommission. Nach Rücksprache mit der zuständigen Ethikkommission wurde deshalb kein formelles Votum als erforderlich angesehen.

Die Durchführung erfolgte in Übereinstimmung mit den ethischen Grundsätzen der Deklaration von Helsinki. Alle Studienteilnehmer wurden mündlich und schriftlich über Ziel, Ablauf und Inhalte der Untersuchung aufgeklärt und gaben ihr schriftliches Einverständnis zur Teilnahme. Die erhobenen Daten wurden pseudonymisiert ausgewertet.

## Datenverfügbarkeitserklärung

Die im Rahmen der vorliegenden Studie erhobenen und analysierten Datensätze sind aufgrund ethischer und datenschutzrechtlicher Bestimmungen nicht öffentlich zugänglich. Sie sind jedoch auf begründete Anfrage beim korrespondierenden Autor und nach Zustimmung der zuständigen Ethikkommission verfügbar.
